# Supplementary figures and images for: Sociality of Cats toward Humans Can Be Influenced by Hormonal and Socio-Environmental Factors: Pilot Study
Source: Animals (Basel). 2022 Dec 30;13(1):146. doi: 10.3390/ani13010146 (PMC9817699; doi:10.3390/ani13010146)

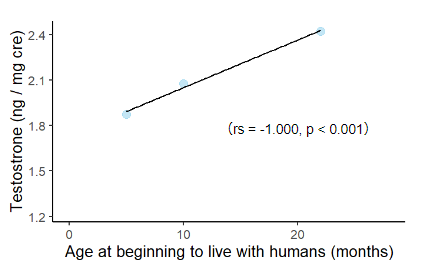

Supplement: Supplementary file 1 [file animals-13-00146-s001.zip › Figure S1.png]
